# Influence of buccal bone lamella defects on hard and soft tissues with submerged and non-submerged healing in immediate implants - an experimental study in minipigs

**DOI:** 10.1186/s40729-025-00607-9

**Published:** 2025-03-12

**Authors:** Christian Mehl, Sönke Harder, Leonie Zenzen, Hendrik Naujokat, Jörg Wiltfang, Yahya Acil, Matthias Kern

**Affiliations:** 1 Department of Prosthodontics, Propaedeutics and Dental Materials, Christian-Albrechts University at Kiel, Arnold-Heller-Strasse 16, Kiel, Germany; 2 Orthodontic practice Dres. Golland, Bahnhofsplatz 7, Chur, 7000 Switzerland; 3 Private Practice, Eichkoppelweg 74, 24119 Kronshagen, Germany; 4 Department of Oral and Maxillofacial Surgery, Christian-Albrechts University at Kiel, Arnold-Heller- Straße 16, 24105 Kiel, Germany

**Keywords:** Implant insertion, Submerged, Non-submerged, Buccal bone defect, Osseointegration, Soft tissue, Hard tissue

## Abstract

**Purpose:**

This study assessed the impact of the buccal bone on hard and soft tissues in submerged and non-submerged immediate implants using a minipig model.

**Methods:**

Sixty-five titanium implants (Camlog Progressive Line) were placed in four minipigs immediately after tooth extraction. All non-submerged (NSM) implants received a mechanically induced buccal bone defect (NSM-BD), whereas the submerged group (SM) was classified as defective (SM-BD) and intact (SM-BI). All bone defects underwent guided bone regeneration (GBR). After four months, the minipigs were sacrificed. Harvested specimens were analysed using histomorphometry and light and fluorescence microscopy. The evaluated parameters included the sulcus (S), implant epithelium (IE), connective tissue (CT), biological width (BW), highest soft tissue point (HSTP), and first hard tissue contact (FHTC).

**Results:**

Of the 65 implants four (6%) were lost, while all remaining implants demonstrated clinical stability (Periotest). Despite GBR failures caused by the pigs’ hay consumption after one week, no significant differences (*p* > 0.5) were observed between SM-BD and NSM-BD in buccal parameters (NSM-BD/SM-BD: S = 0.6 mm, IE = 2.9/2.4 mm, CT = 3.5/3.4 mm, BW = 5.9/5.8 mm). Compared to SM-BI soft-tissue parameters increased in length with reduced buccal bone lamella (SM-BI/SM-BD: S = 0.4/0,6 mm; *p* ≤ 0.04, SM-BI/NSM-BD: IE = 1.8/2.9 mm; *p* ≤ 0.007, SM-BI/SM-BD: CT = 2.5/3.4 mm; *p* ≤ 0.01, BW = 4.0/5.8 mm; *p* ≤ 0.007). The buccal HSTP remained unaffected (*p* > 0.5; (NSM-BD = 1.8 mm, SM-BD = 1.0 mm, SM-BI = 2.0 mm; *p* > 0.5) for all groups.

**Conclusion:**

A buccal bone defect resulted in prolonged S, IE, CT, and BW. However, the aesthetic parameter HSTP did not exhibit significant differences (*p* > 0.5) at the buccal implant site when comparing the SM and NSM healing protocols.

**Graphical Abstract:**

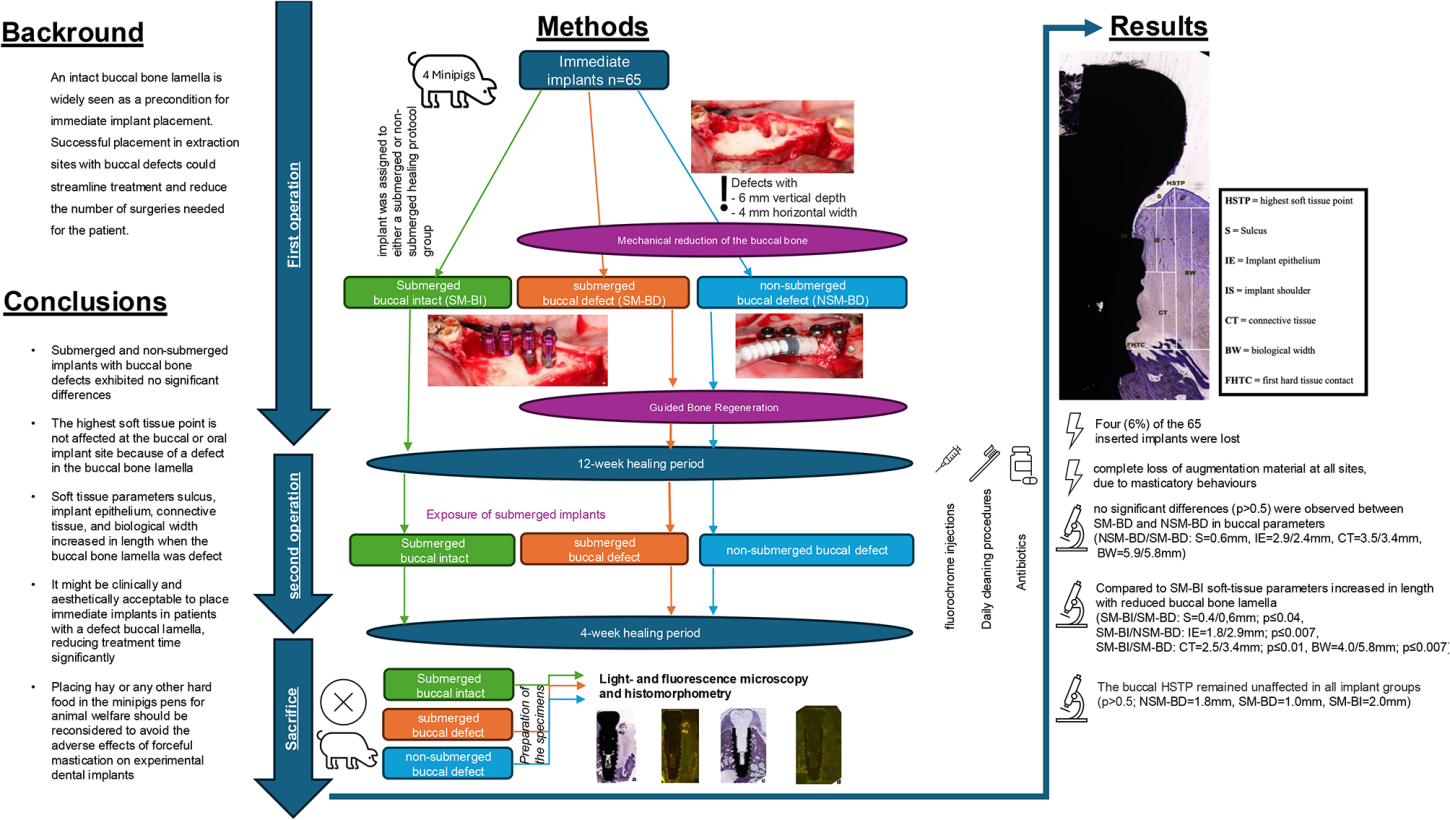

## Background

In modern dental implantology, the quest for optimal results and patient satisfaction has led to advances in surgical techniques [1]. Among these advancements, immediate implant insertion [2], submerged and non-submerged healing protocols [3], and guided bone regeneration (GBR) techniques [4] are pivotal components for shortening treatment duration [5] and ensuring successful implant integration [6].

The treatment procedure for immediate implants involves placement of dental implants in the extraction socket immediately after tooth removal [7]. With comparable survival rates of 95% for immediate implants and 98% for late implants [8], the immediate implant technique offers patients and practitioners the advantage of reduced surgical intervention [5].

Physiological hard and soft tissue changes are inevitable following tooth extraction [9]. In particular, the buccal bone lamella, a thin outer bone structure of the alveolar ridge [10], is vulnerable [11] and undergoes major dimensional changes after extraction [9]. Its function is considered an important component in maintaining the aesthetic soft tissue profile [12] around dental implants. Many animal [13–15] and clinical [16, 17] studies have shown that immediate implantation alone cannot alter the physiological remodelling process [18]. However, the developed technique of GBR [19, 20] has a positive influence [21]. The use of barrier membranes and bone-graft materials [22] facilitates bone regeneration in areas with insufficient volume or density [23].

After successful immediate implantation with GBR, one- or two-stage healing procedures [24] can be used. Two-stage submerged healing protocol completely covers the implant with soft tissues during the initial healing phase [25], implants in the one-stage non-submerged healing protocol remain exposed to the oral environment [26]. Both methods have shown clinically comparable aesthetic outcomes [27] and survival rates [28, 29].

The studies mentioned above demonstrated that the bone is the primary support for the soft tissue around the inserted implants [30]; however, several studies [31–33] have reported good soft tissue results with a defective buccal bone lamella. Practitioner apprehension about compromised aesthetic outcomes during immediate implant placement into defect sockets [34] could prolong treatment. The discrepancies between these studies raise the question of the significance of the buccal bone lamella.

This study aimed to gather histomorphometric data to evaluate the impact of a buccal bone defect on the surrounding hard and soft tissues during immediate implant treatments. GBR was performed to support the bone remodelling processes [35]. The differentiation between submerged and non-submerged healing protocols will demonstrate the soft tissue response when the buccal bone lamella is reduced. Successful immediate implant insertion into buccal defect sockets would streamline treatment planning for practitioners and provide patients with shorter treatment durations and pleasing aesthetics while helping to evaluate the relationship between hard and soft tissues.

## Methods

### Operational planning

**Fig. 1:**
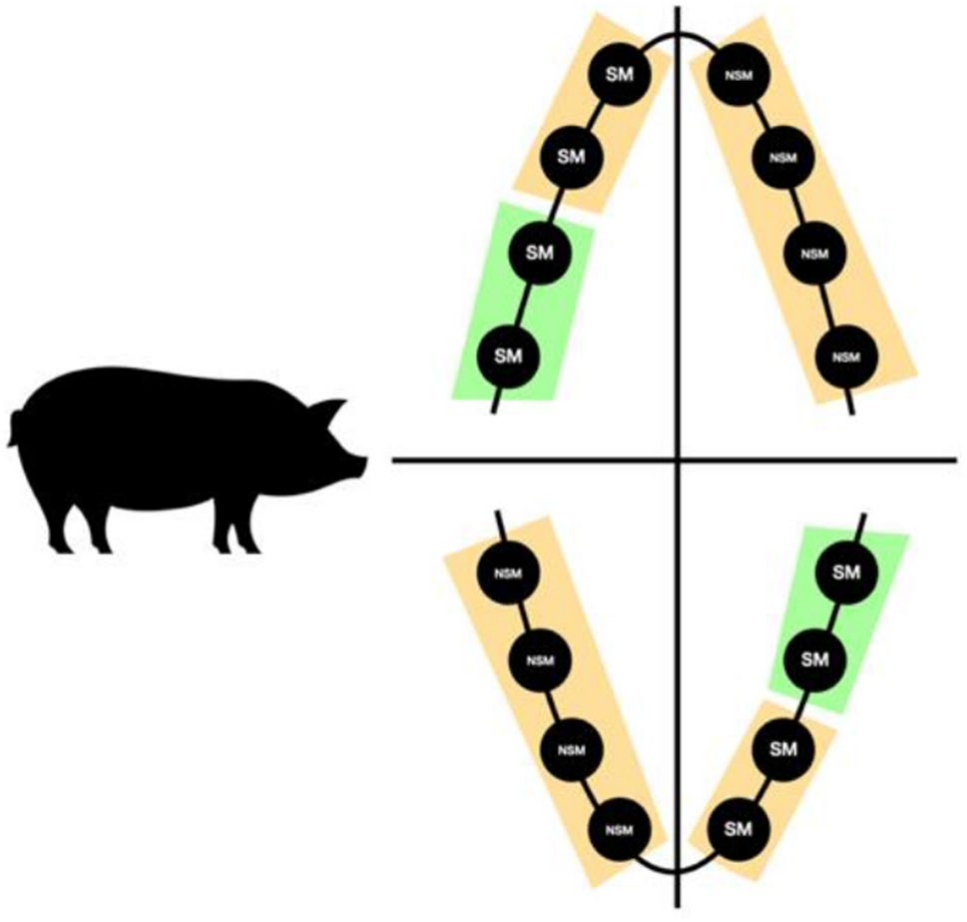
A schematic representation of the insertion protocol based on previous studies utilizing a split-mouth design to compare submerged and non-submerged healing protocols. Submerged (SM) implants were also placed in the anterior region of the jaw. **Orange**: reduced buccal bone; **Green**: intact buccal bone; **SM**: submerged implants; **NSM**: non-submerged implants

Four minipigs (all female, 12 months old, body weight of 48.8 ± 9 kg) were the study subjects. Each animal received eight implants per jaw (Progressive Line; Camlog Biotechnologies, Basel, Switzerland) using a split-mouth design (Fig. 1).

Before implant insertion, each implant was assigned to either a submerged or non-submerged healing protocol group. In the non-submerged group, buccal bone reduction was performed for all implants (NSM-BD). The submerged group (SM) was further subdivided into two categories: those with a defective buccal bone (SM-BD) and those with an intact buccal bone (SM-BI). All implants were inserted bone level. All bone defects measured 6 mm in vertical depth and 4 mm in horizontal width. The defects were fully filled to restore the anatomical contour through the use of guided bone regeneration (GBR) with resorbable membranes (Mem-Lok Pliable, BioHorizons, USA) and a bovine-derived bone graft substitute (MinerOss X-syringe). Consistent with previous studies [36, 37] and to ensure the use of comparable protocols, the experimental duration was extended over 16 weeks, encompassing the interval from implant placement to sacrifice of all study animals.

### Surgical procedures

Table 1 lists the materials used in this study. This study was approved and complied with the European Welfare Act: Experiment Permit V-242-33872/2020 (57 − 7/20). To ensure appropriate husbandry, the minipigs were housed in indoor and outdoor pens and provided food, water ad libitum, and hay for comfort.

**Table 1:** Materials used in this study

Proprietary material	Manufacturer	Type	Lot No.
Camlog Progressive - Line	Camlog Biotechnologies	11 mm × Ø 4.3 mm screw - mounted	0010102333
Gingivaformer PS, wide body	Camlog Biotechnologies	Ø 4.3 mm GH 4.0 mm	0100101677
Healing cap PS, wide body	Camlog Biotechnologies	Ø 4.3 mm GH 4.0 mm	0100101677
MinerOss X	BioHorizons	Bovine Bone Graft In Syringe	BM1CSU18P1
Mem-Lok Pliable	BioHorizons	15 × 20 mm Resorbable Collagen Membrane for Dental Surgery	PRLU19M4
ALTApin Magazine	Camlog Biotechnologies	Titanium pins	0120100906

The minipigs were administered intramuscular injections throughout all surgical interventions. The injections consisted of 4% azaperone (4 mg/kg; Stresnil, Lilly, Germany), 10% ketamine (10 mg/kg; Bremer Pharma, Germany), and 0.5% midazolam (1.8 mg/kg, B. Braun, Germany) to induce sedation. Intubation anaesthesia was performed using a straight Miller laryngoscope (size 4) and a 5.5 mm standard tube (Portex, Kent, United Kingdom) using isoflurane (Isoflurane CP, CP Pharma, Germany).

**Fig. 2:**
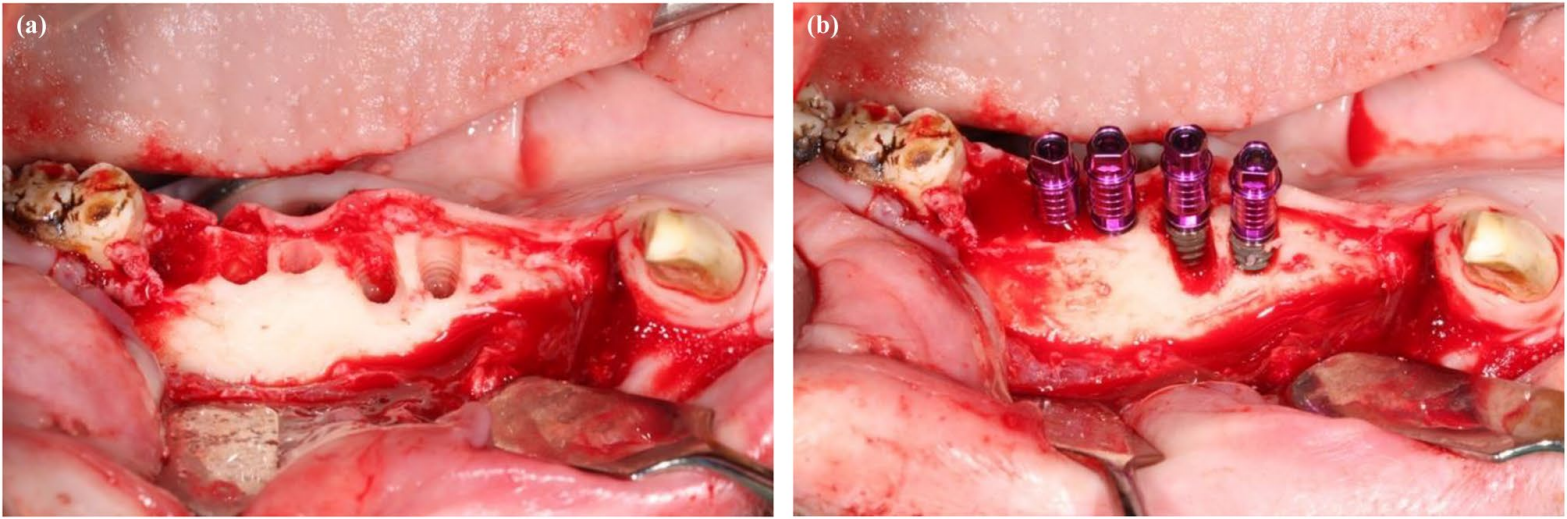
**a** Preparation of the extraction socket for immediate implantation using the Camlog implant drill (Camlog Biotechnologies, Basel, Switzerland) in the following order: two sockets without buccal bone reduction following two implant cavities with mechanical buccal bone reduction. **b** Camlog implants inserted into the mechanically prepared extraction sockets

After sedation was established, the first procedure began with the injection of a local anaesthetic (Ultracain D-S forte, Hoechst, Germany) into all premolars and first molars. After mobilizing the soft tissue and periosteum, the teeth were extracted with meticulous care to minimize additional bone loss. Each implant cavity was prepared according to the manufacturer’s instructions (Fig. 2a). A sterile saline solution (0.9%) was used for cooling throughout the procedure to mitigate the risk of overheating the bone.

Mechanical reduction of the buccal bone was performed across all non-submerged and submerged implants designated before treatment (Figs. 1 and 2a, and 2b). The submerged implants received a cover screw (Camlog Biotechnologies), whereas the non-submerged implants received a transgingival abutment (PS, wide body, Camlog Biotechnologies).

**Fig. 3:**
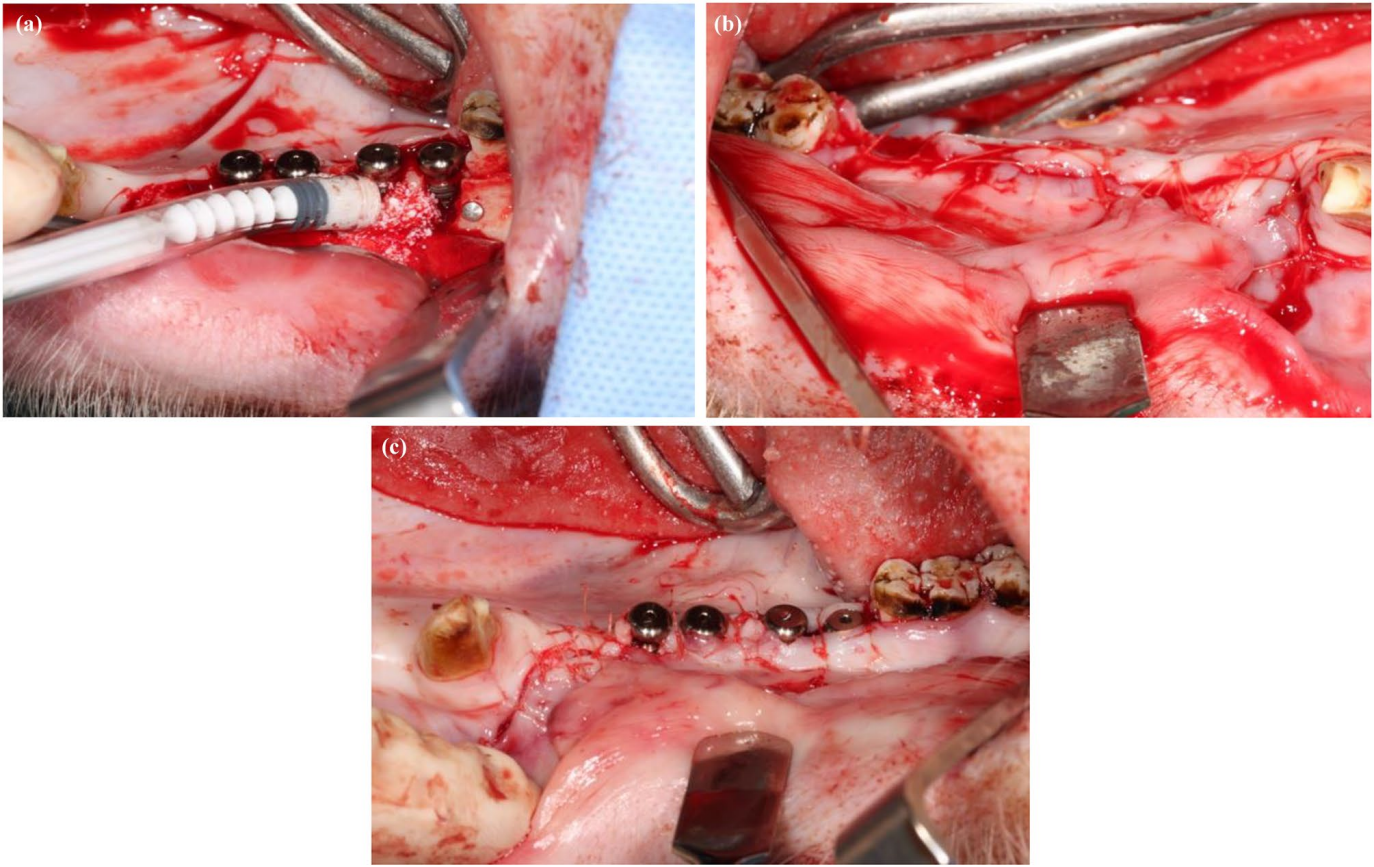
**a** Syringe augmentation with bovine bone substitute material (MinerOss X) for the non-submerged implants. **b** Mucosal closure of the submerged implants. **c** Mucosal closure of the non-submerged implants

GBR was performed with resorbable membranes (Mem-Lok Pliable; BioHorizons, USA) covering all buccal bone defects. Four titanium nails (Camlog Altapin) were used to fix the membranes for each augmentation. Subsequently, the membrane pocket was filled with an allogenic bovine bone graft substitute (MinerOss X syringe; Fig. 3a). No periosteal incisions were performed to minimize flap tension. Each operation was completed by closing the soft tissue using degradable sutures (Vicryl^®^ 3.0, Vicryl^®^ 1.0, Ethicon Inc., Germany) (Fig. 3b and c).

Antibiotics (10 mg/kg body weight, Endofloxacin, 10% Baytril, Bayer, Germany) and analgesics (4 mg/kg BW, Rimadyl, Carprofen, Pfizer, Germany) were administered postoperatively [37, 38].

All minipigs received fluorochrome injections to analyse the bone remodelling processes. The first injection was administered on the seventh postoperative day and then every 2 weeks as follows:

- Xylenol orange 6% in 2% NaHCO_3_ (90 mg/kg body weight) at weeks 1, 3, and 5.
- Calcein blue 1% in 2% NaHCO_3_ (15 mg/kg body weight) at weeks 7 and 9.
- Tetracycline hydrochloride (30 mg/kg body weight) at weeks 11, 13, and 15.

Concurrent with the administration of fluorochrome injections, all minipigs underwent professional dental cleaning procedures targeting both implants and residual teeth. After a 12-week healing period, a second surgical procedure was performed to reveal the submerged implants and affix the transgingival abutment. Subsequently, the osseointegration of all implants was assessed using a Periotest device.

Four weeks later, all minipigs were sacrificed, and the jaws were harvested. Analgesia was achieved through the intramuscular administration of azaperone (2 mg/kg), midazolam (1.8 mg/kg), and 10% ketamine (10 mg/kg). Euthanasia was induced by intravascular injection of 40 mg/kg pentobarbital (Narcoren, Merial, Germany) into the minipigs’ ears. To prevent potential mechanical or thermal damage to the implants during specimen retrieval, a cooling procedure using sterile saline solution (0.9%) was implemented, maintaining a safe distance of at least 1 cm from the implant.

### Preparation of the specimens

Specimens were prepared as described by Mehl et al. [37]. The jaw halves were immediately saturated in freshly mixed 4% paraformaldehyde at 4 °C. The fixative solution was replaced every week. Following a 3-week period, the specimens were dehydrated using an ascending ethanol series with an embedding machine (Type 1.412.00, Pool of Scientific Instruments, Germany). Subsequently, the specimens were embedded in resin at 6 °C and polymerized for 3 days at 37 °C. For histological examination, the technique described by Donath and Breuner (Donath 1985) was used. After polymerization, each jaw was sectioned into individual implant specimens using a Metabo saw (Wiesmoor, Germany). Each implant was further sliced into 100-µm sections in the anterior-posterior direction (Exakt Apparatebau, Germany; Mehl, et al., 2013) to enable microscopic analysis.

### Light and fluorescence microscopy and histomorphometry

**Fig. 4:**
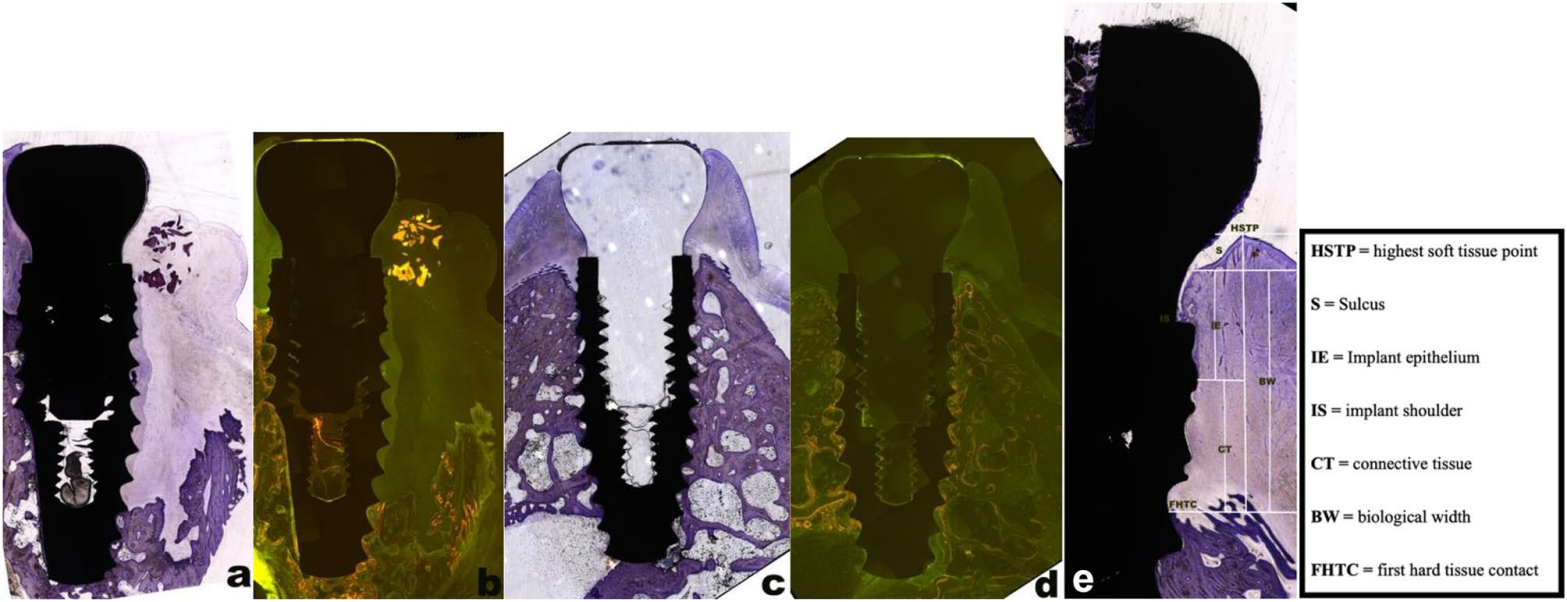
(**a**) Exemplary toluidine blue staining used for histological evaluation with reduced buccal bone lamella (magnification 2.5×). (**b**) shows the fluorescent dye-merged photograph of (**a**) (magnification 2.5×). (**c**) Examples of toluidine blue staining used for histological evaluation of intact buccal bone lamellae (magnification 2.5×). (**d**) shows the fluorescent-dyed merged photograph of (**c**) (magnification 2.5×). (**e**) A detailed exemplification of the histological measurements using the Adobe Photoshop software

Each specimen was polished to a thickness of 50 μm, followed by visual examination under a fluorescence microscope (Mikrophot-FXA; Nikon, Japan) and digital imaging (Q500MC; Leica Cambridge Ltd., England). After imaging, the specimens were stained with toluidine blue solution for 15 min. Adobe Photoshop software was used for histological assessment of the specimens (Fig. 4a-e). The parameters evaluated for each experimental implant group included the sulcus (S), implant epithelium (IE), connective tissue (CT), biological width (BW), highest soft tissue point (HSTP), and first hard tissue contact (FHTC), as shown in Fig. 4e.

### Statistical analysis

The data was analysed using SPSS for Windows software (version 23.0, SPSS, Chicago, IL, USA) and the open-source software R (version 4.3.1, package lme4). All measured parameters showed significant deviations from the normal distribution according to the Shapiro-Wilk test. The study findings were summarized utilizing the first and third quartile medians in mixed linear models. Non-parametric tests such as the Wilcoxon rank-sum test were used. All statistical analyses were conducted at a 95% confidence level.

## Results

A summary of the histological data and the corresponding statistical analyses is presented in Tables 2, 3 and 4. Four (6%) of the 65 inserted implants were lost. All implant failures were observed in the same minipig and localized in the mandibular jaw, which had been treated using the NSM/BD healing protocol. The remaining implants demonstrated clinical stability, as measured using the Periotest. The first week after implant placement revealed a complete loss of augmentation material at all sites. Despite deviations from the protocol, this study was completed. Inflammation was observed in the soft tissue surrounding the implants after two weeks despite strict adherence to weekly professional oral cleansing procedures. The inflammation was more significant in the mandible than in the maxilla. After 10 weeks, the inflammation diminished significantly.

Fluorescence photographs were visually evaluated, and no discernible differences were observed between the implants.

**Table 2:** Comparison of the submerged and non-submerged implants with reduced and intact buccal bone of buccal and oral implant site

Implant Site	Comparison	N	S		IE		CT		BW		HSTP		FHTC	
			P	Median in mm	P	Median in mm	P	Median in mm	P	Median in mm	P	Median in mm	P	Median in mm
Buccal	NSM-BD	25	> 0.5	0.6	> 0.5	2.9	> 0.5	3.5	> 0.5	5.9	> 0.5	1.8	> 0.5	4.1
	SM-BD	17		0.6		2.4		3.4		5.8		1.0		4.1
	SM-BD	17	**≤ 0.04**	0.6	**≤ 0.04**	2.4	**≤ 0.01**	3.4	**≤ 0.007**	5.8	> 0.5	1.0	**≤ 0.0002**	4.1
	SM-BI	16		0.4		1.8		2.5		4.0		2.0		1.5
	SM-BI	16	> 0.5	0.4	**≤ 0.007**	1,8	**≤ 0.02**	2,5	**≤ 0.001**	4,0	>0.5	2,0	**≤ 0.0004**	1,5
	NSM-BD	25		0,6		2,9		3,5		5,9		1,8		4,1
Oral	NSM-BD	27	> 0.5	0.4	> 0.5	3.1	**≤ 0.01**	2.1	> 0.5	5.6	> 0.5	3.2	> 0.5	1.8
	SM-BD	14		0.4		2.5		1.8		4.5		3.3		1.4
	SM-BD	14	> 0.5	0.4	> 0.5	2.5	> 0.5	1.8	> 0.5	4.5	> 0.5	3.3	> 0.5	1.4
	SM-BI	16		0.5		2.4		2.0		4.4		3.3		1.1
	SM-BI	16	> 0.5	0.5	> 0.5	2.4	> 0.5	2.0	> 0.5	4,4	> 0.5	3.2	**≤ 0.04**	1.1
	NSM-BD	27		0.4		3.1		2.1		5,6		3.2		1.8

General linear model. The values were averaged for calculation purposes. Values of P **≤** 0.05 are in bold. SM-BI: submerged with intact buccal; SM-BD: submerged with buccal defect; NSM-BD: non-submerged with buccal defect

**Table 3:** Comparison of buccal and oral implant site of submerged and non-submerged implants with reduced and intact buccal bone lamella

Implant Group	Comparison	N	S		IE		CT		BW		HSTP		FHTC	
			P	Median in mm	P	Median in mm	P	Median in mm	P	Median in mm	P	Median in mm	P	Median in mm
NSM-BD	Buccal	25	> 0.5	0.6	> 0.5	2,9	**≤ 0.0001**	3.5	**≤ 0.001**	5.9	**≤ 0.007**	1.8	**≤ 0.0001**	4.1
	Oral	27		0.4		3,1		2.1		5.6		3.2		1.8
SM-BD	Buccal	17	> 0.5	0.6	> 0.5	2.4	**≤ 0.001**	3.4	**≤ 0.02**	5.8	**≤ 0.03**	1.0	**≤ 0.0002**	4.1
	Oral	14		0.4		2.5		1.8		4.5		3.3		1.4
SM-BI	Buccal	16	> 0.5	0.4	> 0.5	1,7	> 0.5	2.5	> 0.5	4.0	> 0.5	2.0	> 0.5	1.5
	Oral	16		0.4		2,4		2.0		4.4		3.2		1.1

General linear model. The values were averaged for calculation purposes. P **≤** 0.05 are in bold

**Table 4:** Comparison of upper and lower jaws within the implant testing group

Implant Group	Comparison	N	S		IE		CT		BW		HSTP		FHTC	
			P	Median in mm	P	Median in mm	P	Median in mm	P	Median in mm	P	Median in mm	P	Median in mm
SM-BI	U	8	> 0.5	0.4	> 0.5	2.4	> 0.5	2.2	> 0.5	4.5	**≤ 0.02**	3.1	**≤ 0.001**	1.1
	L	8		0.3		1.5		2.5		4.0		1.4		2.8
SM-BD	U	8	> 0.5	0.5	> 0.5	2.2	> 0.5	3.3	> 0.5	5.9	**≤ 0.0001**	2.6	**≤ 0.01**	3.2
	L	9		0.8		2.4		3.4		5.8		0.6		6.3
NSM-BD	U	15	> 0.5	0.5	**≤ 0.003**	3.1	> 0.5	3.0	> 0.5	6.1	**≤ 0.001**	2.3	> 0.5	3.7
	L	10		0.7		2.6		3.9		5.7		1.0		6.5

General linear model. The values were averaged for calculation purposes. Values of P **≤** 0.05 are in bold. U: upper jaw; L: lower jaw

The buccal implant site comparison between the SM-BD and NSM-BD groups revealed no significant differences (*p* > 0.5) across all measured hard and soft tissue parameters. In the SM-BD and NSM-BD groups, only the HSTP was maintained, with no significant difference (*p* > 0.5), compared with the SM-BI group. All other soft-tissue parameters (S, IE, CT, and BW) demonstrated an increase in length when the buccal bone was reduced. Conversely, the FHTC exhibited minimal alterations, maintaining a consistent median length of 4.1 mm, in the NSM-BD and SM-BD groups.

When comparing the oral implant sites, significant differences were observed only in the CT (*p* ≤ 0.01) between the SM-BD and NSM-BD groups. Further comparisons of intact and defect buccal bone revealed no significant differences in the S, IE, CT, BW, HSTP (*p* > 0.5) and FHTC (*p* ≤ 0.04).

Comparisons between the oral and buccal implant sides further corroborated most of the findings mentioned above. Buccal implant sites with bone reduction showed significant differences with increased length on the CT, BW, and FHTC (Table 3). The S and IE showed no significant differences (*p* > 0.5). Only the HSTP indicated a shorter length (NSM-BD: *p* ≤ 0.007; SM-BD: *p* ≤ 0.03) compared to the oral measurements.

A comparative analysis of the maxilla and mandible (Table 4) revealed statistically significant differences in the HSTP and FHTC. The maxilla exhibited a lower FHTC (median: SM-BI 1.1 mm; SM-BD 3.2 mm) and an increased HSTP (median: SM-BI 3.1 mm; SM-BD 2.6 mm), whereas the mandible had a higher FHTC (median: SM-BI 2.8 mm; SM-BD 6.3 mm) and a decreased HSTP (median: SM-BI 1.4 mm; SM-BD 0.6 mm).

## Discussion

Although all GBR sites initially failed, this study demonstrated impressive histological and clinical responses in the soft tissues. In previous minipig studies using dental implants [36, 37], signs of inflammation were observed in the surrounding soft tissues. Despite the adjuvant administration of antibiotics and oral hygiene sessions [37, 39], clinical inflammation occurred in this study. The mandible exhibited heightened inflammatory responses during the healing phase, possibly attributable to the distinctive crushing and grinding masticatory movements [40] that are characteristic of minipigs.

Challenging behavioral manifestations are frequently encountered in clinical investigations involving minipig models [41, 42]. Implant loss [39] is often attributable to intricate oral hygiene practices [43] or masticatory behaviours [40], sometimes leading to the premature cessation of research endeavors [41]. Despite these challenges, minipig models have gained prominence as indispensable tools in dental research [43], because of their comparable bone mineral density [44] and remodelling rate [45] to humans. Animal studies presenting histological examinations of failed GBR accompanied by successfully integrated implants are scarce. In our opinion, the observed GBR failure could be attributed to the minipigs’ preference for masticating hay [41], which was provided ostensibly for physical solace [46], over the prepared soaked granulates. A failure of the GBR procedure, attributable to the structure of the mechanically induced defect or the selected bone substitute material, is highly unlikely based on the daily monitoring of the minipigs. The observations revealed that the mechanical properties of hay lead to a perforation and detachment of all gingival flaps on all GBR sites. Prior to this study it was already duly recognized that providing hay for the animals’ physiological well-being [46] may introduce complexities into clinical implant investigations [41]. However, the ethical imperative of maintaining animal welfare [46] remained paramount throughout the study. Hence, excluding the naturally comforting material from the stables was deemed untenable. Ultimately, although the loss of the GBR sites resulted in non-parametrical data with a lower statistical power the findings of the resulting soft tissue response could be ground breaking for patients receiving immediate implants.

This study included 65 implants, reflective of the typical number of implants used in previous studies [36, 47–49]. The number of implants allocated to each experimental group should be adequate to detect statistically significant differences among the groups [50]. Considering the mechanical impact and the anticipated increased risk of implant loss (Olsen et al., 2004), a larger number of implants was assigned to the non-submerged protocol. After a 12-week period, a total of four implants (6%) with non-submerged healing protocol (NSM-BD) were lost, all from the same minipig which exhibited clinical signs of a periodontal disease. The present results are comparable with those of other minipig studies [39], which reported an average of 6.3 ± 11.4 implants lost. Wound dehiscence is the predominant early healing complication documented in GBR procedures [51, 52]. Although GBR failed, all the remaining implants demonstrated good clinical signs of osseointegration [53].

Consistent with observations from previous investigations [27, 54], the present study revealed no discernible outcome variance between implants with reduced buccal bone and those with submerged or non-submerged healing. The soft-tissue composition surrounding the implant [55] also yielded analogous outcomes between the SM-BD and NSMBD groups. In particular, the peri-implant connective tissue composition exhibited characteristics similar to those of scar tissue [55], with parallel fibre alignment and vascularization orientation across all implant surfaces [56].

Bone remodelling was only observed at the implant surface directly interfacing with the bone, as discerned through histological and visual fluorescence evaluations [36, 37]. There was no evidence of vertical bone remodelling [57] in the mechanically induced bone defects. Only the histological measurements of implants placed in the intact socket (SM-BI) indicated outcomes consistent with the mean tissue measurements reported in previous studies [58, 59]. One potential explanation for this phenomenon can be found in the work of Botticelli et al. [15], which described variations in the surrounding implant tissue as a result of mechanical bone reduction. In addition, the GBR failure during the early healing stages [52] should be considered.

Comparing the buccal implant sites of SM-BD and NSM-BD implants with those of the intact SM-BI, all hard- and soft-tissue parameters in the present study were influenced, except for the HSTP. A comparison between the buccal and oral implant sites and a comparison of the maxilla and mandible revealed differences in the FHTC and HSTP. These findings are consistent with those of previous minipig studies [36, 37]. The results can be attributed to the mechanical reduction of the bone [15] and the distinctive longer oral soft tissue anatomical characteristics of the minipigs presented in this study. Additionally, the mandible exhibits heightened inflammatory responses during the healing phase due to masticatory behaviour [40], which may contribute to a shortened HSTP.

In line with analogous studies [32, 33], postoperative soft tissue improvements were noted with a defect in the buccal bone lamella. HSTP indicates the aesthetic part of the soft tissue covering the implant [34]. Implants were exclusively placed in the posterior region of the jaw. This decision was influenced by the anatomical characteristics of the mini pigs, as the anterior jaw region exhibited insufficient bone thickness for the successful placement of implants. Although soft tissue outcomes [34] have been correlated with the presence of intact buccal bone [30], the present study demonstrated that the HSTP was influenced exclusively when comparing the oral/buccal and mandibular/maxillary implant sites. Considering the buccal implant testing site, the HSTP exhibited no significant influence attributable to the absence of the buccal bone.

## Conclusions

The following can be concluded while considering the study’s limitations:

- Submerged and non-submerged implants with buccal bone defects exhibited no significant differences in any of the measured hard- and soft-tissue parameters at the buccal implant site.
- The highest soft tissue point was not affected at the buccal or oral implant site because of a defect in the buccal bone lamella.
- Soft tissue parameters sulcus, implant epithelium, connective tissue, and biological width increased in length when the buccal bone lamella was reduced.
- The connective tissue around the implants resembles scar tissue in terms of composition, vascularization, and fibre orientation.
- It might be clinically and aesthetically acceptable to place immediate implants in patients with a reduced buccal lamella, reducing treatment time significantly.
- Minipigs presented with a distinctively longer oral soft tissue anatomy with or without a reduced buccal bone lamella.
- The maxilla exhibits a lower functional height of the first hard tissue contact and an increased highest soft tissue point, whereas the mandible shows a higher first hard tissue contact and reduced highest soft tissue point.
- Placing hay or any other hard food in the minipigs’ pens for animal welfare should be reconsidered to avoid the adverse effects of forceful mastication on experimental dental implants. For comparable future studies feeding and housing concepts have to reflect the nature of the experiment to avoid detrimental effects on the study design.

## Data Availability

No datasets were generated or analysed during the current study.

## Abbreviations

S: Sulcus
IE: Implant epithelium
CT: Connective tissue
BW: Biological width
HSTP: Highest soft tissue point
FHTC: First hard tissue point
SM: Submerged
NSM: Non-submerged
SM-BD: Submerged-bone defect
SM-BI: Submerged-bone intact
NSM-BD: Non-submerged-bone defect
GBR: Guided bone regeneration
